# Safety and Efficacy of Fremanezumab for the Prevention of Migraine: A Meta-Analysis From Randomized Controlled Trials

**DOI:** 10.3389/fneur.2020.00435

**Published:** 2020-05-19

**Authors:** Bixi Gao, Nan Sun, Yanbo Yang, Yue Sun, Mingjia Chen, Zhouqing Chen, Zhong Wang

**Affiliations:** Department of Neurosurgery & Brain and Nerve Research Laboratory, The First Affiliated Hospital of Soochow University, Suzhou, China

**Keywords:** fremanezumab, migraine, chronic migraine, episodic migraine, preventive medication, meta-analysis

## Abstract

**Background:** Fremanezumab (TEV-48125) is a fully-humanized immunoglobulin G isotype 2a selective monoclonal antibody that potently binds to calcitonin gene-related peptide (CGRP). It is one of the novel therapeutic drugs for the prevention of migraine, which is one of the most common neurological diseases worldwide. Several controlled trials have been conducted to investigate the safety and efficacy of fremanezumab, however, there is no systematic review of the existing literature has been performed. Hence, in our study, we performed a meta-analysis to investigate the safety and efficacy of fremanezumab for the prevention of migraine.

**Method:** Pubmed (MEDLINE), Embase, and Cochrane Library were searched from January 2001 to August 2019 for randomized controlled trials (RCTs). Five RCTs with 3,379 patients were finally included in our study.

**Result:** We pooled 3,379 patients from 5 RCTs; the primary endpoints were mean monthly migraine and headache days, baseline to week 12. We found that fremanezumab led to a significant reduction in migraine days (*P* < 0.0001) and headache days (*P* < 0.0001) during 12 weeks compared with placebo. Moreover, after using fremanezumab, the risk of at least one adverse event (AE) (*P* = 0.001) and AE related to the trial regimen (*P* = 0.0005) significantly increased compared with the placebo.

**Conclusions:** Fremanezumab showed good efficacy for the prevention of migraine. The administration of fremanezumab can cause some mild adverse events but no serious adverse events.

## Introduction

Migraine is one of the most common neurological disorders, and it is characterized by recrudescent attacks of pulsating headache pain of moderate or severe severity, affecting more than 16% of people worldwide [(1–3). Generally, migraines are divided into episodic and chronic migraine. Episodic migraine is the most common form of migraine lasting fewer than 15 days headache attacks per month (4). However, chronic migraine occurs in ~2% of the population and is defined as headaches that occur at least 15 days per month and lasting at least 3 months (5). Moreover, about 8% of people with episodic migraine have at least 10–14 headache days per month and are at risk of transforming into chronic migraine (at least 15 headache days per month) (6, 7). Both types of migraine can seriously affect the quality life of the patient and in worst cases it may lead to unemployment of the patient (8). Therefore, regardless of types of migraines, timely, and proper preventive measures may benefit patients. The prevention of migraines is not to prevent the illness but to reduce the frequency of migraine attacks (9). The traditional preventive medications on migraines frequently lead to an early suspension of treatment due to their poor efficacy and tolerability in migraine people (10, 11).

The monoclonal antibodies against calcitonin gene-related peptide (CGRP) or its receptor including eptinezumab, fremanezumab, galcanezumab, and erenumab are explicitly developed for the prevention of migraine. Existing research confirmed that the galcanezumab are one of the most effective and safe for migraine prevention (12, 13). Moreover, erenumab and eptinezumab were also proved to be safe and effective in preventing episodic and chronic migraines (14–16). Fremanezumab (TEV-48125) is recognized as a fully-humanized immunoglobulin G isotype 2a selective monoclonal antibody that potently binds to CGRP (17). Fremanezumab blocks CGRP mediated effects by binding to the peptide and it is injected subcutaneously (6, 18, 19). For the prevention of migraine. in previous clinical trials, fremanezumab has been assessed for the safety and efficacy over the 12-week of treatment period. In these clinical trials, fremanezumab exhibited flexible dosing regimens. In chronic migraine, fremanezumab has three dosage regimens during 3 months as follows: 1. 675 mg/225 mg/225 mg; 2. 900 mg/900 mg/900 mg; 3. 675 mg/placebo/placebo. In episodic migraine, fremanezumab also has three dosage regimens: 1. 225 mg/225 mg/225mg; 2. 675 mg/675 mg/675 mg; 3. 675 mg/placebo/placebo. Although the previous five randomized controlled trials concluded that fremanezumab is sufficient to prevent migraines, during this study, we discussed the safety and efficacy of fremanezumab in different populations. Moreover, no systematic review for the existing literature has been carried out before. During our study, we carried out a meta-analysis which included five randomized controlled trials (RCTs), and combined different doses of fremanezumab to evaluate the efficacy and safety of fremanezumab for the prevention of both kinds of migraine.

## Methods

We performed a systematic review and meta-analysis of data from five published studies with the methods described in the PRISMA guidelines (20).

### Search Strategy

Pubmed (MEDLINE), Embase, and Cochrane Library were searched using the following terms: [(“fremanezumab, migraine,”) (“TEV-48125 and migraine”)] until December 2019 to find potentially eligible studies. Besides, we ensured all relevant studies had been included in this meta-analysis by manually screening reference lists from RCTs and systematic reviews.

### Inclusion and Exclusion Criteria

Inclusion criteria were as follows: (a) Study type: RCTs; (b) Language restriction: our study had no language restriction; (c) Participants: patients aged >18 years with chronic or episodic migraine, and were allowed to use preventive medications or not; (d) Intervention: fremanezumab and comparator (placebo); (e) Outcomes: efficacy outcomes including mean monthly migraine days, mean monthly headache days and safety outcomes. Exclusion criteria were as follows: (a) study types: case reports, reviews, retrospective studies, *post-hoc* analyses studies and cohort studies; (b) According to previous RCTs, patients as follows were excluded: patients who used onabotulinumtoxinA during 4 months before screening; patients who used interventions or devices for migraine during 2 months before screening; patients with previous exposure to a monoclonal antibody targeting the CGRP pathway.

### Study Selection and Data Collection

All studies and reference lists of RCTs and reviews of systematic searches in electronic databases were evaluated separately on the mentioned inclusion and exclusion criteria. After carefully assessing and selecting, the basic information of the included trials (first author, title, number of NCT, patient characteristics (age, sex, BMI, migraine classification, etc.), outcome measures were used to extract the data (Table 1).

**Table 1:** Characteristics of the included studies and outcome events.

**Trials**	**Bigal, 2015 (21) (NCT02021773)**	**Bigal, 2015 (18) (NCT02025556)**	**Silberstein et al. (22) (NCT02621931)**	**Dodick et al. (23) (NCT02629861)**	**Ferrari et al. (24) (NCT03308968)**
**Information of the Included Trials**					
Regions	Multicenter, in USA	Multicenter, in USA	Multicenter, in nine countries	Multicenter, in nine countries	Multicenter, in fifteen countries
Phases	IIB/III	IIB/III	III	III	III
**Eligibility Criteria and Study Design**					
Inclusion Criteria	Chronic migraine Age:18-65 years old No more than 2 different preventive medications or interventions/devices used for migraine.	Episodic migraine Age:18-65 years old No more than 1 preventive medication or intervention/device used for migraine.	Chronic migraine Age:18-70 years old Up to 30% patients were permitted to use no more than 1 concomitant preventive medication	Episodic migraine Age:18-70 years old Up to 30% patients were permitted to use no more than 1 concomitant preventive medication	Chronic migraine and Episodic migraine Age: 18-70 years old Failure from 2-4 different preventive medications using
Exclusion Criteria	OnabotulinumtoxinA use more than 6 months before study. Opioids or barbiturate compounds use more than 4 days during the run-in phase.	Opioids or barbiturate compounds use for more than 4 days during the run-in phase	OnabotulinumtoxinA use more than 6 months before study. Opioids or barbiturate compounds use more than 4 days during the run-in phase.	OnabotulinumtoxinA use more than 6 months before study. Opioids or barbiturate compounds use more than 4 days during the run-in phase.	OnabotulinumtoxinA use more than 3 months before study. Opioids or barbiturate compounds use more than 4 days during the run-in phase.
Study Design and The Number of Subjects	PBO/PBO/PBO (*n =* 89) Fremanezumab 675/225/225 mg (*n =* 88) Fremanezumab 900/900/900 mg (*n =* 86)	PBO/PBO/PBO (*n =* 104) Fremanezumab 225/225/225 mg (*n =* 96) Fremanezumab 675/675/675 mg (*n =* 97)	PBO/PBO/PBO (*n =* 371) Fremanezumab 675/PBO/PBO mg (*n =* 375) Fremanezumab 675/225/225 mg (*n =* 375)	PBO/PBO/PBO (*n =* 294) Fremanezumab 675/PBO/PBO mg (*n =* 291) Fremanezumab 225/225/225 mg (*n =* 290)	PBO/PBO/PBO (*n =* 276) Fremanezumab 675/PBO/PBO mg (EM: *n =* 107; CM: *n =* 169) Fremanezumab 225/225/225 mg in EM (*n =* 110) 675/225/225 mg in CM (*n =* 173)
**Outcomes Assessments**					
Primary outcomes	Mean change from headache hours of any severity, baseline to week 12	Mean change from migraine days, baseline to week 12	Mean change from monthly average headache days of at least moderate severity, baseline to week 12	Mean change from monthly average migraine days, baseline to week 12	Mean change from monthly average migraine days, baseline to week 12
Safety outcomes	Serious adverse events, Injection-site reactions, Headache, Infections,etc.	Serious adverse events, Injection-site reactions, Headache, Infections,etc.	At least one adverse event At least one adverse event related to the trial regimen At least one serious adverse event. Injection-site reactions, Infections, Dizziness, Nausea, etc.	At least one adverse event At least one adverse event related to the trial regimen At least one serious adverse event. Injection-site reactions, Infections, Gastrointestinal disorders, etc.	At least one adverse event At least one adverse event related to the trial regimen At least one serious adverse event. Injection-site reactions, Infections, Gastrointestinal disorders, etc.

### Outcome Measures

The primary outcomes included mean monthly headache days, baseline to week 12, and mean monthly migraine days, baseline to week 12. Baseline means the days of headache or migraine of 28 days before the study started. Secondary endpoints included ≥50% reduction in the average number of migraine days per month, mean monthly days with any acute headache medication, baseline to week 12, mean monthly headache days, baseline to week 4 and mean monthly migraine days, baseline to week 4. Among them, a migraine day was defined as a calendar day with at least four consecutive hours of migraine with or without aura (patients recruited after 2018 need to meet ICHD-3 diagnostic criteria, no more than one ICHD-3 migraine criterion missing) (3), or a headache of any duration treated with migraine-specific acute medications (triptans or ergot compounds). A headache day was defined as a calendar day with at least four consecutive hours of headache at least moderate severity. The adverse events included at least one adverse event (AE) or AE related to the trial regimen or serious AE, injection-site reactions, infections and dizziness or nausea. Among them, injection-site reactions included erythema, induration, pain, bruising, paraesthesia, rash, and warmth. Infections included nasopharyngitis, upper respiratory tract infection, influenza, gastroenteritis, and urinary tract infection.

### Subgroup Analysis

According to the characteristics of studies included in the meta-analysis, three different subgroup analyses can be performed as follow: 1. dosage regimens of fremanezumab. 2. types of migraines. 3. preventive medications. Dosage regimens can be divided into monthly and quarterly administration. Patients were divided into CM subgroups and EM subgroups according to their types of migraines. Also, according to the use of preventive medications, patients were also divided into no more than two used subgroup and 2–4 used subgroup. We compared the efficacy and safety of fremanezumab separately in these different subgroups.

### Summary Measures and Synthesis of Results

Review manager 5.3 was used to assess the data. Estimated profit with mean differences and estimated proportions with the risk ratio (mean difference [MD] or relative risk [RR]; 95% confidence interval [CI]) were calculated using a random-effects model. The *I*^2^ statistic was used to estimate the statistical heterogeneity as follows: *I*^2^ < 30% represents “low heterogeneity,” 30% < *I*^2^ < 50% means “moderate heterogeneity” and *I*^2^> 50% means “substantial heterogeneity.” Sensitivity analysis and subgroup analysis were used to explore the stability of the consolidated results. A <0.05 *P*-value was considered to be significant for all analyses, and tests are two-tailed.

### Risk of Bias

The Review Manager 5.3 software was used to create the risk of bias summary in these studies. The Cochrane collaboration uniform criteria were used to assess the risk of bias of RCTs. Selection bias, performance bias, detection bias, attrition bias, reporting bias, and other possible biases were included in the criteria.

## Results

### Search Results

**Figure 1:**
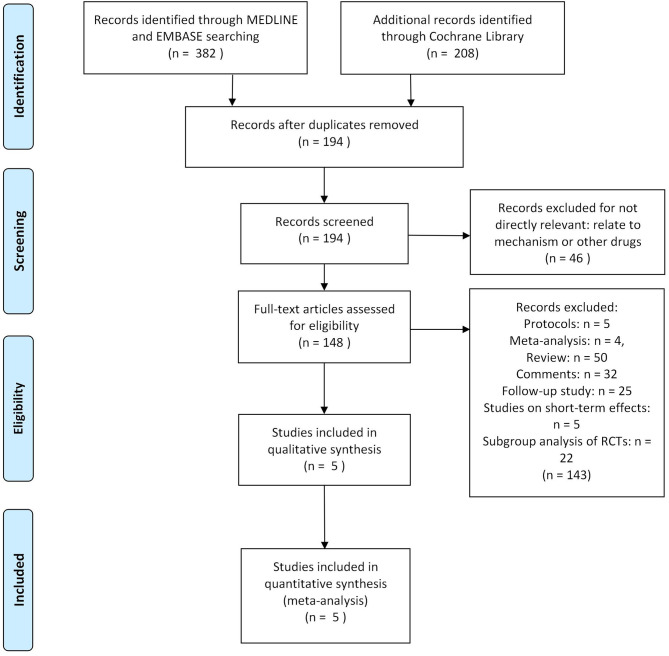
The study search, selection and inclusion process.

Three hundred eighty-two researches and abstracts from pubmed (medline) and embase, and 208 from cochrane library were identified. Three hundred ninety-six researches were removed due to duplicates, and 46 researches were removed because they were not directly relevant to the subject, such as research on other drugs or etiological analysis of migraine, etc. After removing duplicates and uncorrelated titles, 148 of these articles are directly related to the topic of interest. However, among them, 116 articles were excluded because they were protocols, follow-up studies, meta-analyses, reviews, and comments. Besides, five studies on short-term effect (outcomes limited to 4 weeks) and 22 studies on subgroup analysis (such as patients who used triptans before or not) of RCTs were excluded. Thus, at last, these 5 RCTs were included in our study (Figure 1).

### Assessment of Primary Outcomes

**Figure 2:**
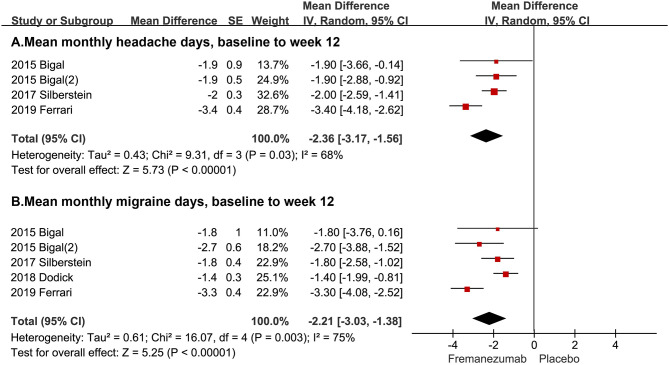
The pooled MD of primary outcomes. The red square indicates the estimated MD for each RCT. The size of red square indicates the estimated weight of each RCT, and the extending lines indicate the estimated 95% CI of MD for each RCT. The black diamond indicates the estimated MD (95% CI) for all patients together. **(A)** Mean monthly headache days, baseline to week 12. **(B)** Mean monthly migraine days, baseline to week 12. Weights are from random-effects analysis. CI, confidence interval; RCT, randomized controlled trial; MD, mean difference.

In this study, two primary outcomes were analyzed included mean monthly headache days, baseline to week 12, and mean monthly migraine days, baseline to week 12. The average mean monthly headache days during 12 weeks in the fremanezumab group is 2.36 days less than that in the placebo group (95% CI, −3.17, −1.56, *P* < 0.0001, Figure 2A). Also, the average mean monthly migraine days during 12 weeks in the fremanezumab group is 2.21 days less than that in the placebo group (95% CI, −3.03, −1.38, *P* < 0.0001, Figure 2B).

### Assessment of Secondary Outcomes

The number of patients who had ≥50% reduction in average number of migraine days per month had a significant increase than placebo (RR = 2.22 95% CI, 1.60, 3.07, *P* < 0.001). The average mean monthly days with any acute headache medication in the fremanezumab group is 2.11 days less than that in the placebo group (95% CI, −3.01, –0.21, *P* < 0.001). The average mean monthly headache days during 4 weeks in the fremanezumab group is 2.65 days less than that in the placebo group (95% CI,−3.63, −1.66, *P* < 0.001). The average mean monthly migraine days during 4 weeks in the fremanezumab group is 2.49 days less than that in the placebo group (95% CI, −3.47, −1.51, *P* < 0.001) (Table 2).

**Table 2:** Other efficacy and safety outcomes.

	**≥50% reduction in average number of migraine days per month**	**Mean monthly days with acute headache medications, baseline to week 12**	**Mean monthly headache days, baseline to week 4**	**Mean monthly migraine days, baseline to week 4**	**Injection-site reactions**	**Infections**	**Dizziness or Nausea**
	**RR, 95% CI**	**MD, 95% CI**	**MD, 95% CI**	**MD, 95% CI**	**RR, 95% CI**	**RR, 95% CI**	**RR, 95% CI**
Bigal (18)	NA	−2.10 (−4.26, 0.06)	−2.40 (−4.16, −0.64)	−2.50 (−4.07, −0.93)	3.21 (1.29, 7.96)	1.03 (0.40, 2.67)	NA
Bigal (21)	1.90 (1.34, 2.68)	−1.70 (−2.88, −0.52)	−1.70 (−2.88, −0.52)	−2.30 (−3.48, −1.12)	1.64 (0.84, 3.22)	1.09 (0.59, 2.04)	0.96 (0.29, 3.20)
Silberstein et al. (22)	2.17 (1.72, 2.74)	−2.10 (−2.69, −1.51)	−2.40 (−3.18, −1.62)	NA	1.12 (1.03, 1.22)	0.90 (0.64, 1.27)	0.93 (0.51, 1.68)
Dodick et al. (23)	1.65 (1.34, 2.03)	−1.30 (−1.69, −0.91)	NA	−1.70 (−2.29, −1.11)	1.26 (1.13, 1.41)	1.06 (0.74, 1.51)	1.11 (0.39, 3.16)
Ferrari et al. (24)	3.99 (2.68, 5.95)	−3.30 (−3.89, −2.71)	−3.80 (−4.58, −3.02)	−3.50 (−4.28, −2.72)	1.24 (0.91, 1.70)	0.94 (0.61, 1.46)	0.83 (0.37, 1.88)
Total	2.22 (1.60, 3.07) *P < * 0.001	−2.11 (−3.01, −1.21) *P < * 0.001	−2.65 (−3.63, −1.66) *P < * 0.001	−2.49 (−3.47, −1.51) *P < * 0.001	1.24 (1.07, 1.43) *P =* 0.003	0.98 (0.81, 1.20) *P =* 0.86	0.93 (0.62, 1.40) *P =* 0.73

### Assessment of Adverse Events

**Figure 3:**
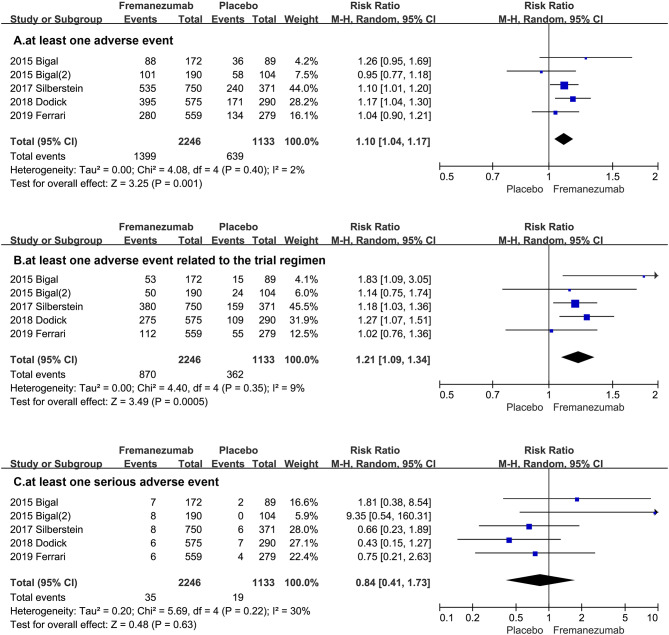
The pooled RR of adverse events. The blue square indicates the estimated RR for each RCT. The size of blue square indicates the estimated weight of each RCT, and the extending lines indicate the estimated 95% CI of RR for each RCT. The black diamond indicates the estimated RR (95% CI) for all patients together. **(A)** at least one adverse event. **(B)** at least one adverse event related to the trial regimen. **(C)** at least one serious adverse event. CI, confidence interval; RCT, randomized controlled trial; RR: risk ratio.

The comprehensive analysis shows that the proportion of patients with at least one adverse event in the fremanezumab group is more than the placebo group (RR = 1.10, 95% CI, 1.04, 1.17, *P* = 0.001, Figure 3A). Then, our statistical analysis suggests that the fremanezumab group is more likely to suffer from adverse events related to the trial regimen (RR = 1.21, 95% CI, 1.09, 1.34, *P* = 0.0005, Figure 3B). However, the proportion of patients with at least one serious adverse event in the fremanezumab group has no significant difference from the placebo group (RR = 0.84 95% CI, 0.41, 1.73, *P* = 0.63, Figure 3C).

We also analyzed several special adverse events, including injection-site reactions, infections and dizziness or nausea. In fremanezumab group, the incidence rate of injection-site reactions is higher than the control group, while the proportion of infections and dizziness or nausea have no significant difference (Injection-site reactions: RR = 1.24, 95% CI, 1.07, 1.43 *P* = 0.003; Infections: RR = 0.81, 95% CI, 0.81, 1.20, *P* = 0.86; Dizziness or Nausea: RR = 0.93, 95% CI, 0.62, 1.40, *P* = 0.73, Table 2).

### Subgroup Analysis

#### Efficacy

According to previous clinical trials, the dosage regimens of fremanezumab were roughly divided into monthly and quarterly administrations. When administrating monthly, the average mean monthly headache days during 12 weeks in fremanezumab group is 2.85 days less than the placebo group (95% CI, −4.32, 1.38, *P* = 0.0001), and the average mean migraine days during 12 weeks in fremanezumab group is 2.27 days less than the placebo group (95% CI, −3.52, −1.01, *P* = 0.0004). When administrating quarterly, the average mean monthly headache days during 12 weeks in fremanezumab group is 2.55 days less than the placebo group (95% CI, −4.02, 1.08, *P* = 0.0007), and the average mean migraine days during 12 weeks in fremanezumab group is 2.00 days less than the placebo group (95% CI, −3.17, −0.83, P = 0.0008). However, there is no difference between monthly and quarterly administration of fremanezumab in efficacy for preventing migraines (headache days: *P* = 0.78, and migraine days: *P* = 0.86).

Patients were divided into chronic migraine subgroups and episodic migraine subgroups according to the characteristics of migraines. In chronic migraine subgroup, the average mean monthly migraine days during 12 weeks in fremanezumab group is 2.43 days less than the placebo group (95% CI, −3.70, −1.17, *P* = 0.0002), and in episodic migraine subgroup, the average mean monthly migraine days during 12 weeks in fremanezumab group is 2.36 days less than the placebo group (95% CI, −3.55, −1.17, *P* = 0.0001). When it comes to another primary outcome, in chronic migraine subgroup, the average mean monthly headache days during 12 weeks in fremanezumab group is 1.99 days less than the placebo group (95% CI, −2.55, −1.43, *P* = 0.0001), and in episodic migraine subgroup, the average mean monthly headache days during 12 weeks in fremanezumab group is 1.90 days less than the placebo group (95% CI, −2.88, −0.92, *P* = 0.0001). However, differences between CM and EM subgroups are not statistically significant in both outcomes (headache days: *P* = 0.88, and migraine days: *P* = 0.93).

The subjects were also divided into two different subgroups based on if they had used no more than two preventive medications or 2–4 kinds of preventive medications. In no more than two preventive medications subgroup, the average mean monthly migraine days during 12 weeks in fremanezumab group is 1.77 days less than the placebo group (95% CI, −2.29 −1.25, *P* = 0.0001), and in 2–4 preventive medications subgroup, the average mean monthly migraine days during 12 weeks in fremanezumab group is 3.30 days less than the placebo group (95% CI, −4.08, −2.52, *P* = 0.0001). When it comes to another primary outcome, in no more than two preventive medications subgroup, the average mean monthly headache days during 12 weeks in fremanezumab group is 1.97 days less than the placebo group (95% CI, −2.45, −1.48, *P* = 0.0001), and in 2–4 preventive medications subgroup, the average mean monthly headache days during 12 weeks in fremanezumab group is 3.40 days less than the placebo group (95% CI, −4.18, −2.62, *P* = 0.0001). What's more, differences between no more than two preventive medications subgroup and 2–4 preventive medications subgroup are significant in both outcomes (headache days: *P* = 0.002, and migraine days: *P* = 0.001) (Table 3).

**Table 3:** Subgroup analyses of efficacy and safety outcomes.

	**Efficacy**				**Safety**					
	**Mean monthly headache days, baseline to week 12**		**Mean monthly migraine days, baseline to week 12**		**At least one adverse event**		**At least one adverse event related to trial regimen**		**At least one serious adverse event**	
	**MD, 95% CI**	***P-*value**	**MD, 95% CI**	***P-*value**	**RR, 95% CI**	***P-*value**	**RR, 95% CI**	***P-*value**	**RR, 95% CI**	***P-*value**
**Dose regimens**										
Monthly	−2.85 (−4.32, 1.38)	0.0001	−2.27 (−3.52, −1.01)	0.0004	1.22 (1.01, 1.47)	0.04	1.36 (1.12, 1.65)	0.002	0.70 (0.33, 1.47)	0.35
Quarterly	−2.55 (−4.02, −1.08)	0.0007	−2.00 (−3.17, −0.83)	0.0008	1.34 (1.11, 1.62)	0.002	1.30 (1.07, 1.58)	0.007	0.47 (0.20, 1.09)	0.08
Subgroup differences	*P =* 0.78		*P =* 0.86		*P =* 0.48		*P =* 0.74		*P =* 0.48	
**Types of migraine**										
CM	−1.99 (−2.55, −1.43)	0.0001	−2.43 (−3.70, −1.17)	0.0002	1.12 (1.03, 1.21)	0.01	1.37 (0.91, 2.06)	0.13	0.93 (0.36, 2.37)	0.87
EM	−1.90 (−2.88, −0.92	0.0001	−2.36 (−3.55, −1.17)	0.0001	1.08 (0.89, 1.31)	0.44	1.25 (1.07, 1.47)	0.005	1.58 (0.06, 41.14)	0.78
Subgroup differences	*P =* 0.88		*P =* 0.93		*P =* 0.75		*P =* 0.69		*P =* 0.76	
**Preventive medications**										
No more than 2 used	−1.97 (−2.45, −1.48)	0.0001	−1.77 (−2.29, −1.25)	0.0001	1.12 (1.04, 1.20)	0.003	1.23 (1.11, 1.36)	0.0001	0.94 (0.35, 2.53)	0.91
2–4 used	−3.40 (−4.18, −2.62)	0.0001	−3.30 (−4.08, −2.52)	0.0001	1.04 (0.90, 1.21)	0.58	1.02 (0.76, 1.36)	0.91	0.75 (0.21, 2.63)	0.65
Subgroup differences	*P =* 0.002		*P =* 0.001		*P =* 0.42		*P =* 0.22		*P =* 0.78	

### Safety

Monthly and quarterly fremanezumab had no significant difference in safety (at least one AE: *P* = 0.48; at least one AE related to the trial regimen: *P* = 0.74; at least one serious AE: *P* = 0.48). However, no matter which kinds of dosage regimens, the proportion of patients with at least one adverse event in fremanezumab group is more than the placebo group (Monthly: RR = 1.22, 95% CI, 1.01, 1.47 *P* = 0.04; Quarterly: RR = 1.34, 95% CI, 1.11, 1.62 *P* = 0.002). Also, two dosage regimens increased the risk of AE related to the trial regimens (Monthly: RR = 1.36, 95% CI, 1.12, 1.65 *P* = 0.002; Quarterly: RR = 1.30, 95% CI, 1.07, 1.58 *P* = 0.007). However, both of two dosage regimens did not increase the risk of serious AE (Monthly: RR = 0.70, 95% CI, 0.33, 1.47 *P* = 0.35; Quarterly: RR = 0.47, 95% CI, 0.20, 1.09 *P* = 0.08).

In CM subgroup, the proportion of patients with at least one adverse event in fremanezumab group is more than placebo group (RR = 1.12, 95% CI, 1.03, 1.21 *P* = 0.01). However, the proportion of patients with AEs related to trial regimen (RR = 1.37, 95% CI, 0.91, 2.06 *P* = 0.13). or serious AE (RR = 0.93, 95% CI, 0.36, 2.37 *P* = 0.13) is no more than placebo group. In EM subgroup, the proportion of patients with at least one adverse event related to trial regimen in fremanezumab group is more than placebo group (RR = 1.25, 95% CI, 1.07, 1.47 *P* = 0.005) However, the proportion of patients with at least one AE (RR = 1.08, 95% CI, 0.89, 1.31 *P* = 0.75) or serious AE (RR = 1.58, 95% CI, 0.06, 41.14 *P* = 0.76) is no more than placebo group.

In no more than two preventive medications subgroup, the proportion of patients with at least one adverse event (RR = 1.12, 95% CI, 1.04, 1.20 *P* = 0.003) or AEs (RR = 1.23, 95% CI, 1.11, 1.36 *P* = 0.0001) related to the trial regimen in fremanezumab group is more than the placebo group. Nevertheless, t serious AE in fremanezumab and placebo group had no difference (RR = 0.94, 95% CI, 0.35, 2.53 *P* = 0.78). In the 2–4 preventive medications subgroup, no matter which safety outcomes in fremanezumab and placebo group had no difference (Table 3).

### Risk of Bias in Included Studies

**Figure 4:**
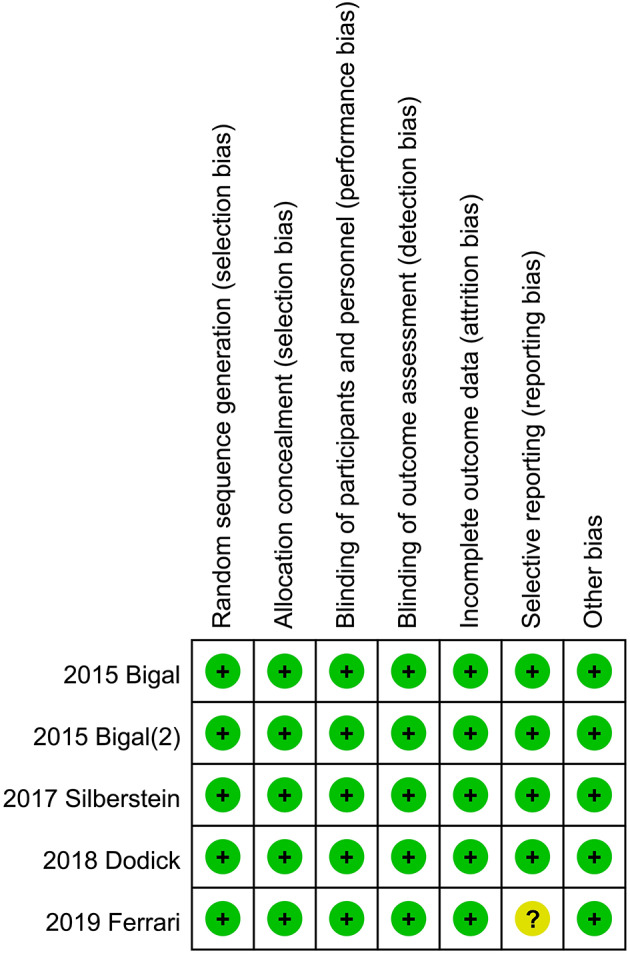
Risk of bias: a summary table for each risk of bias item for each study.

The independent risk of bias of the five included RCTs is shown in Figure 4 in detail. The risk for selective reporting bias is unclear in Ferrari's study in 2019. In addition to the measure, other studies have low risks of bias (Figure 4).

## Discussion

Our meta-analysis included 3,379 patients from 5 multicenter RCTs (18, 21–24) which provided high levels of clinical reliability to assess the safety and efficacy of fremanezumab for the prevention of chronic and episodic migraine. According to the results of our meta-analysis, fremanezumab was more significantly effective for the prevention of CM or EM vs. placebo. By comparing two primary outcomes, we found that the use of fremanezumab was associated with a reduction in the mean monthly migraine and headache days during 12 weeks. Subsequently, the use of fremanezumab caused reduction in acute headache medication use and increased proportion of 50% responders. Also, in short-term effects, fremanezumab showed significantly positive effects. Regarding treatment safety, the use of fremanezumab may cause several mild trial-related or unrelated AEs, especially injection-site reactions. Nevertheless, the use of fremanezumab was not associated with severe AEs.

During previous clinical trials, fremanezumab exhibited had flexible dosage regimens. In our study, we combined different dosage regimens of fremanezumab to analyze the efficacy and safety. Moreover, we performed a subgroup analysis of monthly and quarterly administration. The monthly administration was defined as 675 mg (in CM) or 225 mg (in EM) in the first month and 225 mg in the second and third month. Quarterly administration was defined as 675 mg in the first month and placebo in the second and third month. After analysis, we found that both dosage regimens were effective for preventing migraines. We were unable to determine that which dosage regimen was more effective because both dosage regimens achieve effective blood concentration (25). The *European Headache Federation* found that monthly fremanezumab had a higher quality of evidence than quarterly fremanezumab (16). However, our results of meta-analysis were inconsistent with Sacco's study. It is for the reason that we added Ferrari's study into our meta-analysis, and achieved a different conclusion. In previous studies, headache days were used as the primary outcome for EM, while migraine days were used as the primary outcome for CM. Therefore, we identified mean monthly migraine days and headache days, baseline to week 12, as primary outcomes. The definitions of a migraine day and a headache day were the same as those in previous clinical trials. On the other hand, the use of acute headache medication and the proportion of 50% responders were assessed as secondary outcomes. In our meta-analysis, not only long-term effects but also the short-term effects were assessed. Therefore, we identified mean monthly migraine days and headache days, baseline to week 4, as secondary outcomes. After analysis, we found that in all efficacy outcomes, fremanezumab was more effective than the placebo. Nonetheless, as previous studies reported, fremanezumab indeed showed a significant effect on preventing migraine attacks.

However, it was found that the heterogeneity of the results was high in assessing primary outcomes, and was recorded with >50%. One of the possible reasons was that we pooled the results of phase II and phase III trials (26). Bigal's two studies were in phase IIB, but the other three studies were in phase III. Another possible reason of the heterogeneity probably related to the existence of patients with different characteristics during these clinical trials. Previous studies were performed separately for chronic migraine (CM) or episodic migraine (EM). However, study carried out by Ferrari et al. (24) combined CM and EM for analysis. From another perspective, subjects from four RCTs used not more than two preventive medications. Nevertheless, patients in Ferrari's study failed from 2 to 4 preventive medications. Therefore, due to the heterogeneity >50% and the different study designs, we used the random-effect model to analyze our data. Consequently, we performed a subgroup analysis to assess the efficacy of fremanezumab in patients with different types of migraines and different preventive medications using. After subgroup analysis, we found that in both CM and EM, fremanezumab had a significant effect on preventing migraines. The effectiveness of fremanezumab was not associated with the types of migraines. Whereas, irrespective of patients using number of preventive medications, fremanezumab was proved to be effective in preventing migraines. Furthermore, fremanezumab showed a better effect on patients who used more preventive medications. Meanwhile, the *European Headache Federation* recommended patients who have failed at least two preventive medications using fremanezumab to prevent migraines (16). Our results had proved the expert's opinion. However, the results depend on the favorable results of Ferrari's study, researches are still needed to prove this opinion. Although using preventive medications may affect the effectiveness of fremanezumab, existence of placebo and nocebo phenomena in each subgroup cannot be overlooked.

Placebo phenomena can be defined with the patients who use an inactive agent for achieving better outcomes in anticipation of better healing (27). Whereas, nocebo phenomena is defined for the patients with negative expectations who consider that treatment may harm them by achieving unfavorable outcomes (28, 29). In our meta-analysis, the reduced mean monthly migraine days caused by fremanezumab over placebo is 2.43 days (CM) and 2.36 days (EM) during 12 weeks. The reduced mean monthly headache days caused by fremanezumab over placebo is 1.99 days (CM) and 1.90 days (EM) during 12 weeks. The study carried out by Kokoti found that placebo was more useful in EM patients than CM patients (30). Therefore, this tiny gap of the efficacy in CM and EM may be caused by the placebo phenomenon. Nocebo phenomena may affect the patients who used 2–4 preventive medications. When being treated by placebo, patients who failed from multiple preventive medications achieve a weak effect due to the negative expectations. However, the use of fremanezumab was noticeable for preventing migraines, leading to significant differences in comparing fremanezumab and placebo. On the other hand, patients who failed from 2 to 4 preventive medications had low expectations, and they were more likely to achieve better results. Nonetheless, whether the use of preventive medications affects the effects of fremanezumab is still controversial and unresolved. However, after subgroup analysis, the heterogeneity of data in each group was significantly reduced, which also showed that the sources of heterogeneity were the type of migraine and the use of preventive medications.

### Safety

In the past, many studies have comprehensively described the safety of fremanezumab. The study carried out by Bigal and Silberstein concluded that the use of fremanezumab was generally safe (31, 32). However, results regarding security were not consistent in these RCTs. After our meta-analysis, we found that the fremanezumab does indeed do not cause some serious adverse events or even death except can cause some mild adverse events, especially injection-site reactions. Previous studies showed that fremanezumab works outside the brain and not directly on the central nervous system (33). Therefore, fremanezumab may cause some adverse events outside the central nervous system such as injection-site reactions and infections, instead of adverse events in central nervous system. For this reason, the incidence of adverse events shows no significant difference between patients with CM and EM. On the other hand, patients who used multiple preventive drugs developed some tolerance to different kinds of medications and avoided some adverse events. Therefore, the incidence of mild adverse events was lower in patients who used 2–4 preventive medications before. During this study, we comprehensively evaluated the benefits and risks of using fremanezumab, and our results showed that, the use of fremanezumab for preventing migraines is feasible due to fremanezumab does not cause serious adverse events. However, evaluations of these studies on adverse events were limited to 12 weeks after the first dose. Therefore, whether fremanezumab will produce long-term (more than 1 year) adverse events, still needs further observation.

### Limitations

Our meta-analysis also has few limitations. As interventions in the included studies were all fremanezumab and placebo, and therefore we can only conclude that fremanezumab is more effective than the placebo in the prevention of migraines. Previous studies concluded that low to high quality of evidence to use eptinezumab, erenumab, fremanezumab, and galcanezumab for preventing migraines (16). However, this conclusion is not obtained by directly comparing different kinds of CGPR monoclonal antibodies. Although patients in these studies used more or less preventive medications before, all these studies lack a horizontal comparison of fremanezumab with other preventive measures. More studies are needed in future to compare whether fremanezumab is more effective than other preventive medications. Considering different dosage regimens, since patients in Bigal's two different studies in 2015 were not divided into monthly and quarterly administration, a meta-analysis of these two dosing regimens only included three studies. Therefore, the accuracy of the results needs further verification. Also, more studies are needed for assessing monthly and quarterly administration. Moreover, the subjects of these five RCTs were roughly 40–45 years old non-Hispanic and non-Latino white women with a BMI of about 25–27. Although our analysis found the efficacy of fremanezumab for the prevention of migraine is effective in these patients, in order to prove that the scope of application of fremanezumab can be more extensive, more researches on migraine patients of different characteristics are still needed.

## Conclusion

Fremanezumab exhibited good efficacy and safety for the prevention of migraine. Fremanezumab was useful for preventing migraine attacks measured by mean monthly migraine and headache days during 12 weeks after drug ingestion. Irrespective of types of migraines or failures from preventive medications, fremanezumab showed a better effect than the placebo. Fremanezumab has a possible better efficacy in patients with more failures from preventive medications. The administration of fremanezumab may cause some mild adverse events especially injection-site reactions, but it is not related to the significant increase in some serious adverse events.

## Data Availability Statement

All data generated or analyzed during this study are included in this review.

## Author Contributions

ZW was the principal investigator. BG and NS designed the study and developed the analysis plan. MC and YS analyzed the data and performed meta-analysis. BG, YY, and NS contributed in writing of the article. ZC and ZW revised the manuscript and polish the language.

## Conflict of Interest

The authors declare that the research was conducted in the absence of any commercial or financial relationships that could be construed as a potential conflict of interest.

## Abbreviations

CGRP: calcitonin gene-related peptide
RCT: randomized controlled trial
CM: chronic migraine
EM: episodic migraine
NCT: national clinical trial
AE: adverse events
MD: mean difference
RR: relative risk
CI: confidence interval.
